# Diverse Roles of Eph/ephrin Signaling in the Mouse Lens

**DOI:** 10.1371/journal.pone.0028147

**Published:** 2011-11-29

**Authors:** Catherine Cheng, Xiaohua Gong

**Affiliations:** School of Optometry and Vision Science Program, University of California, Berkeley, California, United States of America; Hungarian Academy of Sciences, Hungary

## Abstract

Recent genetic studies show that the Eph/ephrin bidirectional signaling pathway is associated with both congenital and age-related cataracts in mice and humans. We have investigated the molecular mechanisms of cataractogenesis and the roles of ephrin-A5 and EphA2 in the lens. Ephrin-A5 knockout (-/-) mice often display anterior polar cataracts while EphA2(-/-) lenses show very mild cortical or nuclear cataracts at weaning age. The anterior polar cataract of ephrin-A5(-/-) lenses is correlated with multilayers of aberrant cells that express alpha smooth muscle actin, a marker for mesenchymal cells. Only select fiber cells are altered in ephrin-A5(-/-) lenses. Moreover, the disruption of membrane-associated β-catenin and E-cadherin junctions is observed in ephrin-A5(-/-) lens central epithelial cells. In contrast, EphA2(-/-) lenses display normal monolayer epithelium while disorganization is apparent in all lens fiber cells. Immunostaining of ephrin-A5 proteins, highly expressed in lens epithelial cells, were not colocalized with EphA2 proteins, mainly expressed in lens fiber cells. Besides the previously reported function of ephrin-A5 in lens fiber cells, this work suggests that ephrin-A5 regulates β-catenin signaling and E-cadherin to prevent lens anterior epithelial cells from undergoing the epithelial-to-mesenchymal transition while EphA2 is essential for controlling the organization of lens fiber cells through an unknown mechanism. Ephrin-A5 and EphA2 likely interacting with other members of Eph/ephrin family to play diverse functions in lens epithelial cells and/or fiber cells.

## Introduction

The lens is comprised of a monolayer of epithelial cells that covers the anterior hemisphere of bulk elongated fibers, wrapped by a basement membrane called the lens capsule. Lifelong lens growth depends upon a small population of epithelial cells located slightly anterior to the equator in what is known as the circumferential germinative zone. Epithelial cells in the germinative zone continuously proliferate and differentiate into elongating fiber cells at the lens equator [1], [2]. The majority of anterior epithelial cells, also known as central epithelial cells, remain mitotically inactive and stay in close contact with underlying elongating fiber cells via the apical interface [3], [4]. The epithelial-fiber cell interaction exists until the elongating fiber cells reach the anterior suture where the tips of opposing elongating fibers meet each other and detach from the anterior epithelial cells [5]. Thus, the spatial and temporal regulation of epithelial cells is essential for regulating lens growth and homeostasis [6], [7].

Eph/ephrin bidirectional signaling, in which Eph receptors mediating forward signaling in one cell while ephrin ligands initiating reverse signaling in the adjacent cell, has emerged as one of the key cell-cell contact-dependent pathways that coordinate not only developmental processes but also normal physiology and homeostasis of mature organs [8], [9]. The Eph family of receptor tyrosine kinases includes 16 different members, divided into EphA (1 to 10) and EphB (1 to 6) kinases. The ephrin family of ligands consists of ephrin-A (1 to 5) and ephrin-B (1 to 4 and 6). EphA receptors preferentially bind glycosyl-phosphatidylinositol (GPI)-anchored ephrin-A ligands while EphB receptors bind transmembrane ephrin-B ligands. Each receptor interacts with multiple ligands and vice versa. In addition, cross interactions between EphA and ephrin-B or EphB and ephrin-A can also occur [10], [11]. The complementary or overlapping expression pattern of Ephs and ephrins suggests diverse functions of Eph/ephrin signaling in tissue development and in maintaining tissue homeostasis [12].

Altered Eph/ephrin signaling can lead to a variety of diseases in humans [13]. Recent studies report that ephrin-A5 knockout (-/-) mice develop cataracts with variable severity and incomplete penetrance [14], and EphA2 mutations lead to age-dependent cortical cataracts in humans and mice [15], [16], [17], [18], [19]. Cataract formation in ephrin-A5(-/-) and EphA2(-/-) lenses is associated with the disruption of fiber cell organization and the alteration of adhesion junctions [14], [16]. Upregulation of Hsp25 was detected in the EphA2(-/-) lenses [16]. However, the functional roles of ephrin/Eph signaling remain unclear in the lens. It is also unknown how variable and age-dependent cataracts develop in either mutant mouse line [14], [16].

In order to minimize the possibility that lens phenotypes might be manipulated by different mouse strain backgrounds [20], [21], we have characterized the lenses of ephrin-A5(-/-) and EphA2(-/-) mice, mainly in the C57BL/6J strain background without the CP49 deletion reported in other mouse strains [22], [23], [24]. We have found that ephrin-A5 is important for maintaining anterior lens epithelial cells and that EphA2 is essential for the organization of lens fiber cells.

## Results

### Different lens phenotypes between ephrin-A5 and EphA2 knockout mice

**Figure 1:**
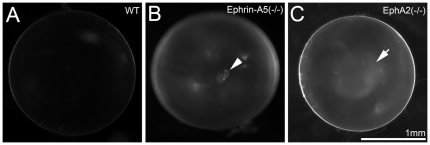
Different types of cataracts occur in ephrin-A5(-/-) and EphA2(-/-) mice. Photos of enucleated fresh lenses from P21 wild-type (WT) (A), ephrin-A5(-/-) (B) and EphA2(-/-) (C) mice. A representative ephrin-A5(-/-) lens photo shows an obvious cataract at the anterior pole (arrowhead in B). A representative EphA2(-/-) lens displays mild nuclear opacity (arrowheads in C). Scale bar, 1mm.

Like wild-type (WT) lenses (Figure 1A), both ephrin-A5 and EphA2 heterozygous knockout (+/-) mice had normal lenses (data not shown). Ephrin-A5(-/-) and EphA2(-/-) mice, maintained mainly in the C57BL/6J strain background with wild-type Bfsp2 (or CP49) genes, develop congenital or age-related cataracts with incomplete genetic penetrance that is consistent with previous reports [14], [16]. Moreover, postnatal day 21 (P21) ephrin-A5(-/-) mice often developed anterior cataracts with mild opacities at the anterior polar region (Fig. 1B). Besides cortical cataracts [16], EphA2(-/-) mice sometimes displayed mild nuclear opacities at P21 (Figure 1C). Thus, both ephrin-A5 and EphA2 are important for lens transparency. The phenotypic differences between ephrin-A5(-/-) and EphA2(-/-) mice suggest that ephrin-A5 and EphA2 likely have diverse functions in the lens.

### Ephrin-A5 is important for maintaining anterior epithelial cells

**Figure 2:**
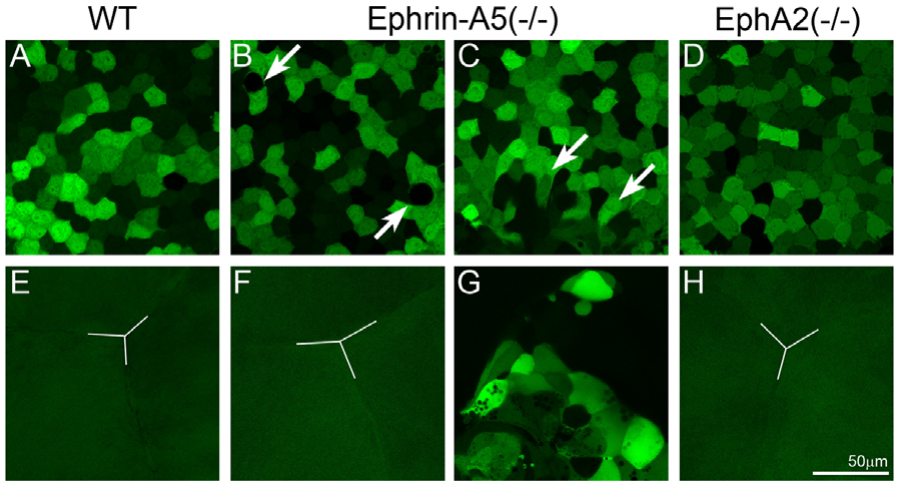
Confocal images of the anterior region of GFP+ wild-type (WT), ephrin-A5(-/-) and EphA2 lenses. The WT lens shows typical mosaic GFP expression pattern in the central epithelium (A) and the Y-shape suture (Y-line) of underlying fiber cells (E). An ephrin-A5(-/-) lens displays morphological changes in a few epithelial cells (arrows in B), and the other ephrin-A5(-/-) lens shows a cluster of aberrant cells underneath the central epithelial cells (arrows in C). In addition, mislocalized aberrant cells are apparent in underlying fiber cell layers without the normal Y-shaped suture (G). The EphA2 (-/-) lens has normal central epithelial cells (D) and anterior Y-shaped suture (H). Scale bar, 50 µm.

To elucidate the cellular mechanism of cataract formation in these knockout mice, a GFP-transgene was bred into both ephrin-A5 and EphA2 knockout mice for morphological examination of lens epithelial and fiber cells in GFP-positive (GFP+) living lenses by using a laser confocal microscope [25], [26]. A GFP+ WT lens showed mosaic GFP expression pattern of typical cuboidal anterior epithelial cells and underlying fiber cells with a Y-shaped suture (Figure 2A and 2E). GFP fluorescent images revealed that all ephrin-A5(-/-) lenses had altered anterior epithelial cells (Figure 2B and 2C) with either a normal Y-suture (Figure 2F) or with a cluster of aberrant cells in the fiber cell layer (Figure 2G). We confirmed that the appearance of the aberrant cell cluster was directly associated with the anterior polar cataract of ephrin-A5(-/-) lenses (arrowhead, Figure 1B). Unlike that in ephrin-A5(-/-) lenses, anterior epithelial cells and the underlying Y-suture in the EphA2(-/-) lens appeared normal (Figure 2D and 2H).

**Figure 3:**
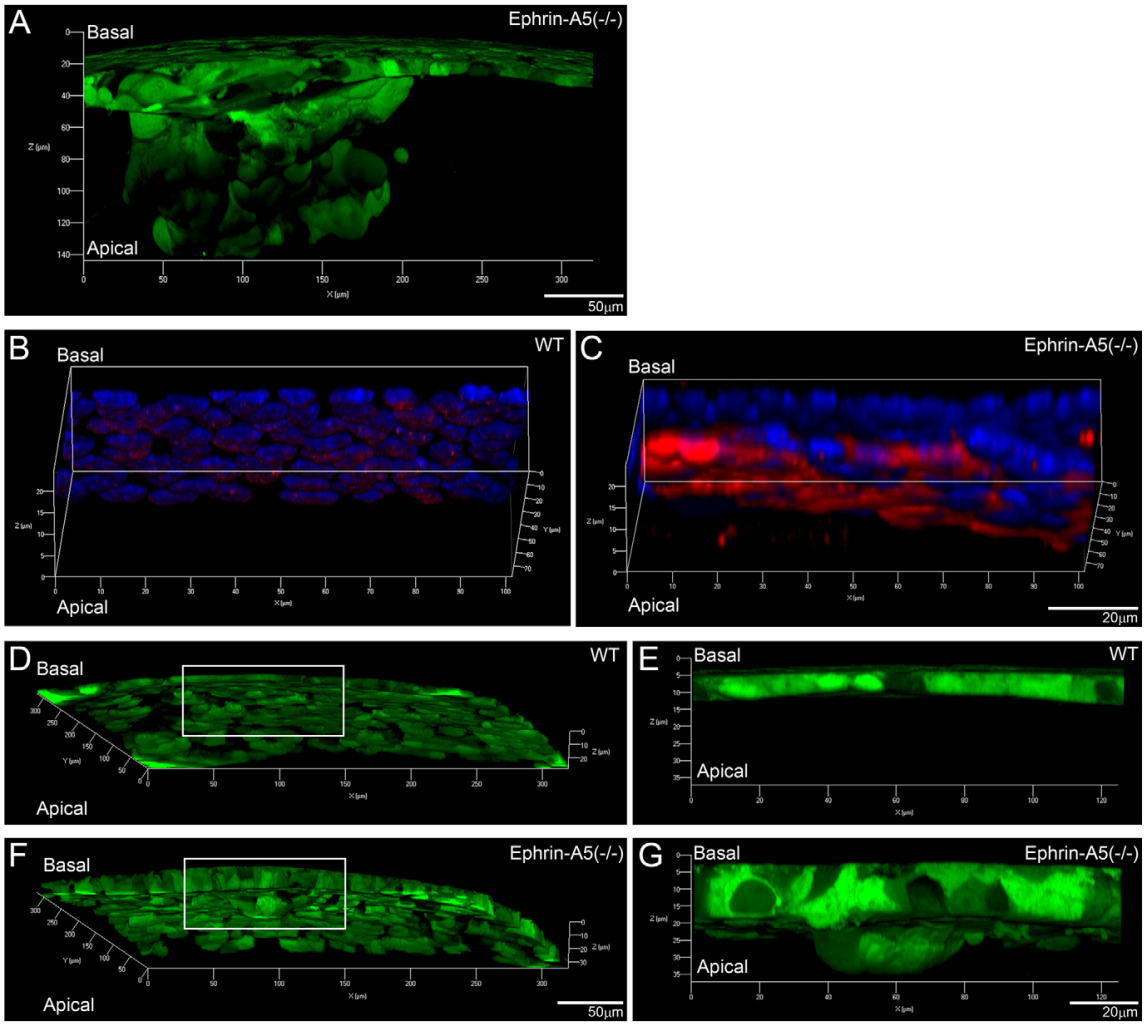
Characterization of lens anterior polar regions in GFP+ WT and ephrin-A5(-/-) lenses. Three-dimensional reconstruction of a P21 GFP+ ephrin-A5(-/-) lens reveals a large cluster of aberrant cells underneath anterior epithelial cells (A). Scale bar, 50 µm. Immunostaining of α-SMA (red) in anterior epithelial cells from lens capsule flat mounts of P21 WT (B) and ephrin-A5(-/-) lenses (C). Only ephrin-A5 (-/-) anterior epithelial cells that are abnormally clustered in the fiber cell layer are obviously positive for α-SMA. Scale bar, 20 µm. Three-dimensional reconstruction of the anterior polar region of P14 GFP+ WT (D) and ephrin-A5(-/-) lenses (F). Scale bar, 50 µm. Enlarged views of anterior epithelial cells in D and F are the boxed areas in panels E and G. Scale bar, 20 µm.

Three-dimensional reconstruction of P21 GFP+ ephrin-A5(-/-) lenses revealed aberrant cells mislocalized into the lens fiber cell layer and disrupted the anterior suture region (Figure 3A). In addition, alpha smooth muscle actin (α-SMA), a marker for mesenchymal cells, was detected in these mislocalized cells (Figure 3C). The mislocalized anterior cell cluster penetrated into the underlying fiber cells and could be detected in ephrin-A5(-/-) lenses as early as P14 (Figure 3F and 3G). Therefore, anterior epithelial cells of ephrin-A5(-/-) lenses undergo epithelial-to-mesenchymal transition (EMT) at the anterior pole region.

### Ephrin-A5(-/-) lens epithelial cells display disrupted distribution of E-cadherin and β-catenin

**Figure 4:**
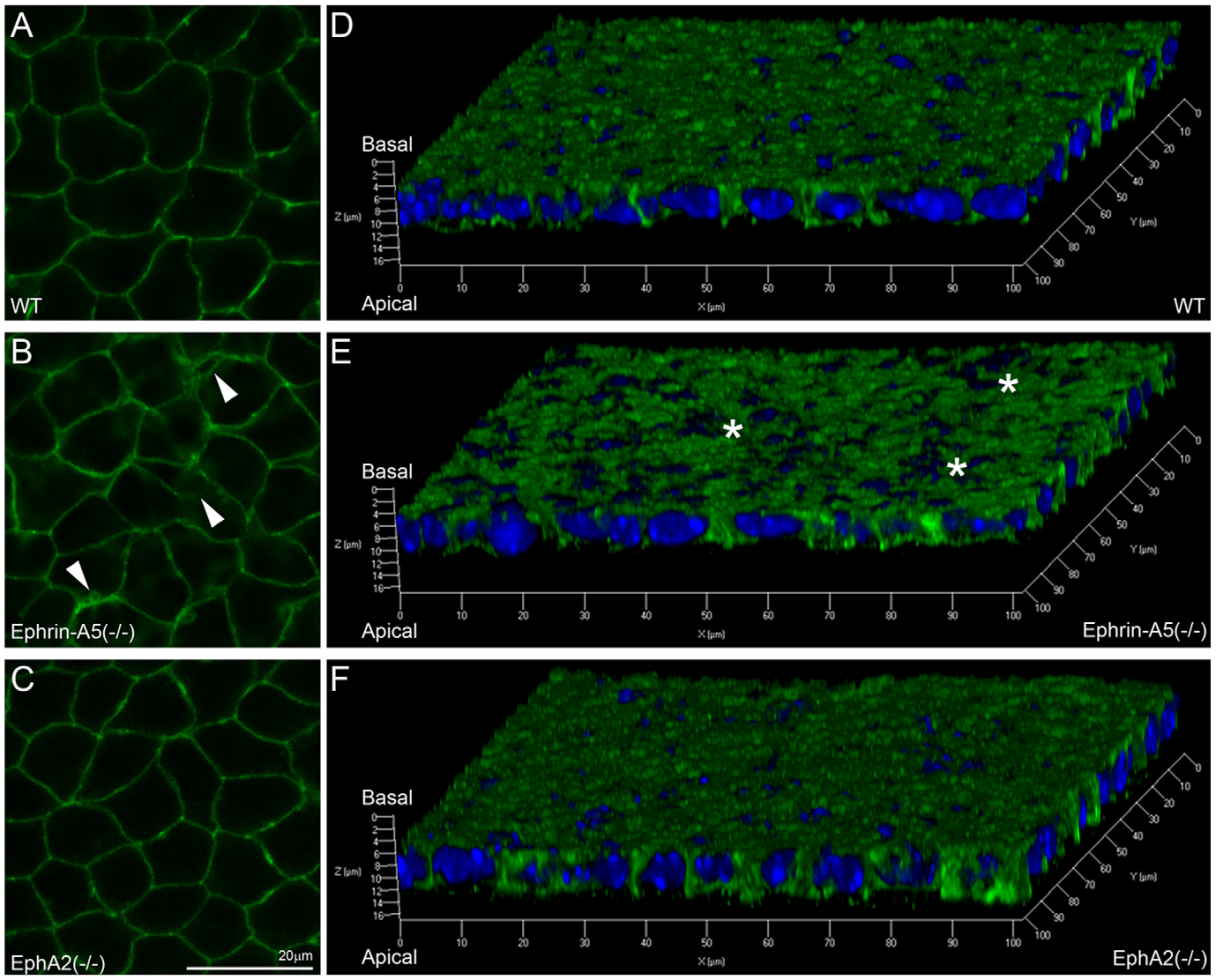
E-cadherin distribution in WT, ephrin-A5(-/-) and EphA2(-/-) lens capsule flat mounts. Fluorescent images reveal normal staining signals of E-cadherin in WT and EphA2(-/-) anterior epithelial cells (A and C) but alterations in the ephrin-A5(-/-) anterior epithelium (B, indicated by arrowheads). Three-dimensional reconstructions of z-stack images labeled for E-cadherin (green) and DAPI (blue, nuclei) of lens epithelial cells from P21 WT, ephrin-A5(-/-) and EphA2(-/-) lens capsule flat mounts (D, E and F). There is a notable disruption of E-cadherin staining in ephrin-A5(-/-) anterior epithelial cells as compared to those in WT and EphA2(-/-) cells. Scale bar, 20 µm.

Cadherin junctions are known to be important for maintaining characteristic features of lens epithelial cells and fiber cells [27], [28]. Lens epithelial cells utilize the E-cadherin while fiber cells express N-cadherin [29]. Fluorescent confocal images of lens capsule flat mounts revealed that E-cadherin proteins were evenly distributed at the membranes of WT and EphA2(-/-) anterior epithelial cells (Figure 4A and 4C) but were abnormally localized at the membranes of ephrin-A5(-/-) anterior epithelial cells (Figure 4B). Three-dimensional images, reconstructed from fluorescent z-stacks of E-cadherin staining, indicated that only ephrin-A5(-/-) epithelial cells show unevenly distributed E-cadherin proteins (Figure 4E, asterisks) when compared to WT and EphA2(-/-) epithelial cells (Figure 4D and 4F).

**Figure 5:**
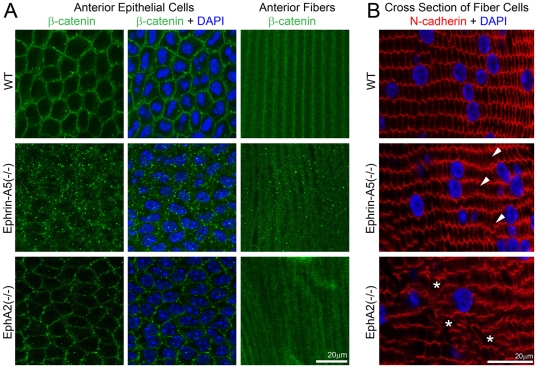
Beta-catenin distribution in anterior epithelial cells and underlying lens fibers and N-cadeherin localization in lens frozen sections. Immunostaining of lens capsule flat mounts shows cell membrane distribution of β-catenin proteins in both anterior epithelial cells and fiber cells of a P21 WT lens (A). Membrane association of β-catenin remains unchanged in EphA2(-/-) anterior lens epithelial cells, but β-catenin proteins form substantial small aggregates in both anterior epithelial cells and fiber cells of ephrin-A5(-/-) lenses (A). Scale bar, 20 µm. N-cadherin is localized at the cell boundaries of hexagonal shaped lens fibers in the WT lens section (B). In the ephrin-A5(-/-) lens section, the majority of fiber cells show normal localization of N-cadherin proteins except select abnormal fiber cells (B, arrowheads). In the EphA2(-/-) lens section, the distribution of N-cadherin proteins is severely altered with some areas lacking N-cadherin staining (B, asterisks). Scale bar, 20 µm.

We further examined the distribution of total β-catenin proteins by immunostaining lens capsule flat mounts. Beta-catenin, known to be an essential signaling component during lens development [30] and in the regulation of cadherin junctions [14], [28], [31], [32], displayed uniformly distributed signals in cell membranes of both anterior epithelial cells and fiber cells of the P21 WT lens (Figure 5A). However, only punctate β-catenin signals were present in ephrin-A5(-/-) anterior epithelial and fiber cells. In contrast, the membrane-associated β-catenin was normal in EphA2(-/-) anterior lens epithelial cells and noticeably diffused distribution of β-catenin proteins appeared in EphA2(-/-) fiber cells (Figure 5A).

We further compared N-cadherin protein distribution in fiber cells of WT, ephrin-A5(-/-) and EphA2(-/-) lens sections (Figure 5B). N-cadherin was localized at the cell boundaries of hexagon-shaped lens fibers. Only select fiber cells showed altered N-cadherin protein distribution in the ephrin-A5(-/-) lens section. But EphA2(-/-) lens fiber cells displayed either severely altered distribution or a lack of N-cadherin proteins. These results indicate that ephrin-A5 is important for the regulation of β-catenin and E-cadherin in lens epithelial cells while EphA2 is needed for normal localization of β-catenin and N-cadherin in lens fiber cells.

### Ephrin-A5 and EphA2 are mainly segregated in the lens

**Figure 6:**
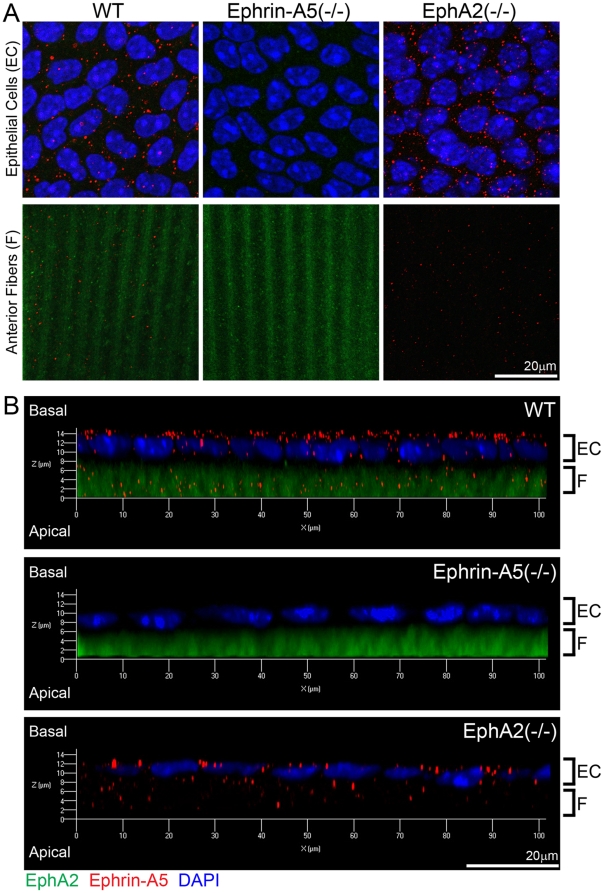
Localization of ephrin-A5 and EphA2 in the lens. Double immunolabeling of ephrin-A5 (red) and EphA2 (green) with DAPI staining (blue, nuclei) of anterior lens epithelial and fiber cells from lens capsule flat mounts of P21 wild-type (WT) and ephrin-A5(-/-), EphA2(-/-) mice (A). Side views of z-stack reconstructions of anterior epithelial cells with underlying fiber cells of P21 WT, ephrin-A5(-/-), EphA2(-/-) lens capsule flat mounts reveal that ephrin-A5 proteins (red) show punctate signals mostly at the lateral and apical sides of lens epithelial cells (EC), and EphA2 proteins (green) show diffused signals in fiber cells (F) (B). Scale bar, 20 µm.

In order to further elucidate molecular basis for the diverse functions between ephrin-A5 and EphA2 in the lens, we examined whether ephrin-A5 and EphA2 proteins were colocalized in lens epithelial cells and/or newly differentiating fiber cells by using immunohistochemical analysis on lens capsule flat mounts. Punctate signals of ephrin-A5 proteins were detected in both anterior epithelial cells and underlying fiber cells of WT and EphA2(-/-) samples, but not in those of the ephrin-A5(-/-) sample (Figure 6A). EphA2 was detected weakly at the cell membrane in epithelial cells and strongly at the membranes of fiber cells of wild-type and ephrin-A5(-/-) samples (Figure 6A). Three-dimensional reconstructions of anterior epithelial cells with underlying fiber cells reveal that ephrin-A5 proteins were mostly present at the lateral and apical sides of lens epithelial cells while EphA2 proteins were predominantly present in fiber cells (Figure 6B). No apparent co-localization of ephrin-A5 and EphA2 staining was observed in either epithelial or fiber cells. Thus, these data demonstrate that segregated protein distribution between ephrin-A5 and EphA2 likely contribute to their diverse functions in lens epithelial and fiber cells.

## Discussion

This work reveals that changes in lens anterior epithelial cells contribute to the cataract formation in ephrin-A5(-/-) mice. A loss-of-function of ephrin-A5 causes lens epithelial cells to undergo epithelial-to-mesenchymal transition (EMT) at the anterior pole region. These clustered mesenchymal cells migrate into fiber cell layers at the anterior suture area to cause anterior cataracts in ephrin-A5(-/-) lenses. EMT has been demonstrated by the expression of α-SMA and the disruption of β-catenin protein distribution at the cell membrane of ephrin-A5(-/-) epithelial cells. Moreover, altered E-cadherin protein distribution is detected in ephrin-A5(-/-) anterior epithelial cells. Conditional knockout of E-cadherin in the lens causes microphthalmia, loss of lens epithelial cells and EMT due to a gradual loss of ZO-1 and β-catenin in lens epithelial cells between embryonic day 16.5 and P14 [27]. Similarly, the loss of β-catenin in the lens causes abnormal E-cadherin expression and increased epithelial cell proliferation resulting in EMT [31], [33]. EMT in the ephrin-A5(-/-) lenses is likely a consequence of disruption of β-catenin signaling and E-cadherin junctions. Thus, ephrin-A5 signaling is essential for maintaining anterior epithelial cells in a monolayer probably by regulating β-catenin-mediated signaling and E-cadherin-mediated adhesion junctions.

At the current stage, we do not know which Eph receptor interacts with ephrin-A5 to prevent abnormal EMT in the lens epithelial cells. Only EphA2 is detected in mouse lens fiber cell membranes by MudPIT proteomics analysis in a recent paper [34]. Our RT-PCR analysis of all members of the ephrin and Eph protein family shows the expression of ephrin-As (A1, A3, A4 and A5), ephrin-Bs (B1, B2 and B3), EphA2, EphA3, EphA4, EphB2, EphB3, EphB4 and EphB6 in lens epithelial cells while expression of only ephrin-A5, EphA2, EphA3, EphA4, EphB4 and EphB6 is present in lens fiber cells (our unpublished data). Thus, it is possible that ephrin-A5 interacts with EphA2 in lens fiber cells. But it is premature to speculate about the possible partners for either ephrin-A5, EphA2 and other Ephs and ephrins in lens epithelial cells. Since EMT does not occur in EphA2(-/-) lenses, EphA2 is unlikely to be the receptor that interact with ephrin-A5 to prevent EMT in lens epithelial cells. It is known that disruption of other Eph/ephrin signaling has been shown to cause abnormal EMT in cardiac development. The appropriate control of EMT is necessary for normal development of the atrioventricular valves. Loss of ephrin-A1, a ligand for EphA3, leads to thickened aortic and mitral valves in mice caused by excessive EMT [35]. Interestingly, EphA3(-/-) mice have decreased EMT, which leads to cardiac abnormalities [36]. These data indicate that EphA3/ephrin-A1 signaling is required for regulating normal EMT in developing cardiac tissue. Thus, ephrin-A5 must interact with another Eph receptor to regulate the properties of lens epithelial cells.

Unlike the role of ephrin-A5 in the lens, EphA2 seems to be essential for the organization of fiber cells probably by regulating N-cadherin-mediated functions. A disruption of N-cadherin is observed in EphA2(-/-) lens fiber cells. N-cadherin is known to be important for differentiation and elongation of lens fiber cells [27], [28]. Previous studies report that N-cadherin/actin complexes form a hexagonal lattice at basal ends of elongating fiber cells, and myosin and integrin β1 may be needed for the migration of the ends of elongating fiber cells [37]. However, mechanisms for how EphA2 regulates the distribution of N-cadherin remain unclear. It is unknown whether a loss of EphA2 and/or the disruption of N-cadherin lead to the change in β-catenin distribution in EphA2(-/-) lens fiber cells. Elevated HSP25 level was detected in EphA2(-/-) lenses [16]. The mechanisms for controlling fiber cell organization and regulating stress responses in the lens are also not well understood.

Unlike a previous study that reported that ephrin-A5(-/-) lenses displayed severely disorganized and rounded fiber cells with altered distribution of N-cadherin [14], we found disrupted fiber cells in limited regions of ephrin-A5(-/-) lenses. Altered distribution of N-cadherin seems to be associated with only select altered fiber cells, but not with organized fiber cells. It is possible that the limited changes of N-cadherin in the fiber cells are due to the role of ephrin-A5 in lens fiber cells and/or an indirect consequence of aberrant EMT in the ephrin-A5(-/-) lenses. However, due to a potential differences of mouse strain backgrounds of ephrin-A5(-/-) mice between the previous study (mouse strain was not stated) and this work as well as variable cataracts among different ephrin-A5(-/-) lenses, it is difficult to speculate the causes for this discrepancy in the severity of lens fiber cell changes. Intricate interactions among different Eph/ephrin pairs in the lens will likely be involved in the variable cataract phenotype in these knockout mice.

In summary, besides functioning in lens fiber cells, ephrin-A5 is essential for maintaining lens central epithelial cells. EphA2 is important for the organization of lens fiber cells. An obvious segregation of ephrin-A5 and EphA2 proteins in lens epithelial cells suggests that other ephrins and Eph receptors likely interact with ephrin-A5 or EphA2 for their diverse functions. Ephrin-A1 is also known to be utilized in lens epithelial cells [16]. Our unpublished RT-PCR results show the expression of many other ephrins and Ephs in the lens. Thus, it will be interesting to further investigate the partners of ephrin-A5 and EphA2 that control the properties of lens epithelial cells and/or the organization of fiber cells. Ephrin-A5 and EphA2 knockout mice are very useful models to further study the regulatory mechanisms of EMT and fiber cell organization, respectively.

## Materials and Methods

### Mice and lens imaging

Mouse care and breeding were performed according to an animal protocol (protocol#: R280-1211BC) approved by the Animal Care and Use Committee at University of California, Berkeley and the ARVO Statement for the Use of Animals in Ophthalmic and Vision Research. The ephrin-A5(-/-) mice [38], a generous gift from Dr. David A. Feldheim at University of California Santa Cruz, have been maintained mainly in the C57BL/6J background. The EphA2(-/-) mice were acquired from The Jackson Laboratory. Both ephrin-A5(-/-) mice and EphA2(-/-) mice were further crossed with C57BL/6J mice for two generations, and the mutant offspring, with normal Bfsp2 (or CP49) genes verified by PCR, were used to breed the mutant mouse lines in this work. Intercross of ephrin-A5(+/-) mice generated ephrin-A5(+/+), (+/-) and (-/-) littermates, and mating of EphA2(-/-) mice produced EphA2(+/+), (+/-) and (-/-) littermates. A standard PCR method [39] was used for genotyping tail DNA isolated from ephrin-A5 mice. The following primers are used for ephrin-A5 genotyping, common: TCCAGCTGTGCAGTTCTCCAAAACA, WT: ATTCCAGAGGGGTGACTACCACATT and knockout: AGCCCAGAAAGCGAAGGAGCAAAGC. These primers produced a 513 base pair knockout band and a 397 base pair wild-type band [38]. The EphA2(-/-) mice were genotyped according to the method provided by The Jackson Laboratory. The following primers were used for Bfsp2 (or CP49) genotyping, C57BL/6 forward: CGCTCTGGGTCTCGCATGAG, 129/SV forward: CAGTCATGTGGTTCTGGAAG and common reverse: CAGCATTATCTACCGTGGTCTGGAG. These primers produced a 205 base pair C57BL/6 wild-type band and a 347 base pair 129/SV mutant band [24]. For imaging, mouse lenses were dissected from enucleated eyeballs, immediately immersed in PBS at 37°C and imaged under a Leica MZ16 dissecting scope using a digital camera.

### Imaging of GFP-positive living lenses

GFP-positive (GFP+) knockout mice were generated by intercrossing ephrin-A5(-/-) mice or EphA2(-/-) mice with GFP transgenic mice [40]. A UV lamp was used to screen GFP+ mice. GFP+ heterozygous mice were mated with non-GFP heterozygous mice to produce GFP+ WT, heterozygous and homozygous mutant mice with one copy of the GFP transgene for image analysis of lens cells.

Fresh intact GFP lenses were dissected immediately before imaging from enucleated eyeballs. Images of lens epithelial and fiber cells with mosaic GFP expression pattern were collected using a Zeiss LSM700 confocal microscope. Lenses were maintained in DMEM medium on the stage of the confocal microscope. To examine the surface characteristics and morphology of GFP+ epithelial cells, z-stack images of lens epithelium were collected with 0.5 µm z-steps. ZEN 2010 software was used to analyze the monolayer of anterior epithelial cells and create three-dimensional reconstructions.

### Immunohistochemistry

Immunostaining of lens frozen sections: Mouse eyes were fixed with fresh 4% paraformaldehyde in phosphate-buffered saline (PBS) for 30 minutes, then washed three times with PBS and soaked overnight in 30% sucrose in PBS. Samples were then processed and sectioned with a Cryostat 1900 (Leica, Germany) using a standard frozen-section method [39]. Lens sections (∼10 µm thick) were stained with mouse anti-N-cadherin (Invitrogen, Carlsbad, CA) primary antibodies overnight at 4°C followed by incubating with fluorescent secondary antibodies (Jackson ImmunoResearch Laboratories, West Grove, PA) for 2 hour at room temperature. Slides were then mounted with DAPI VectorShield mounting medium (Vector Laboratories, Inc., Burlingame, CA). A Zeiss LSM700 confocal microscope was used to examine immunostained lens sections.

Immunostaining of flat mount lens epithelial cells: Mouse lens capsule flat mounts were prepared using a protocol previously described for rat lenses [41]. Briefly, fresh lenses were dissected from enucleated eyeballs. Lenses were immediately fixed for 45 seconds in ice cold methanol. Then the lens capsules (with lens epithelial cells and some cortical fiber cells) were dissected from the fiber mass with radial cuts in 1X PBS. Lens capsules were then placed in a blocking solution (3% bovine serum albumin, 3% normal goat serum and 0.3% Triton X-100 or 10% normal donkey serum and 0.3% Triton X-100) for 1 hour at room temperature. Lens capsules were stained with rat anti-E-cadherin (Invitrogen), rabbit anti-β-catenin (Cell Signaling Technology, Beverly, MA), goat anti-EphA2 (R&D Systems, Minneapolis, MN) and/or rabbit anti-ephrin-A5 (Invitrogen) primary antibody overnight at 4°C and then were washed with 1X PBS 3 times for 5 minutes per wash. Then lens capsules were stained with an appropriate fluorescent secondary antibody (Jackson ImmunoResearch Laboratories) for 2 hours at room temperature. Samples were washed with 1X PBS 4 times for 5 minutes per wash, then quickly dipped in ddH_2_O and flattened and mounted with DAPI VectorShield mounting medium (Vector Laboratories, Inc.). Z-stack images were collected by a Zeiss LSM700 confocal microscope, and three-dimensional images were reconstructed from z-stack data collected at 0.38 µm steps using ZEN 2010 software. Staining was repeated at least three times, and representative results are shown.

## References

[1] Yamamoto N, Majima K, Marunouchi T (2008). A study of the proliferating activity in lens epithelium and the identification of tissue-type stem cells.. Med Mol Morphol.

[2] Piatigorsky J (1981). Lens differentiation in vertebrates. A review of cellular and molecular features.. Differentiation.

[3] Zampighi GA, Eskandari S, Kreman M (2000). Epithelial organization of the mammalian lens.. Exp Eye Res.

[4] Kuszak JR, Novak LA, Brown HG (1995). An ultrastructural analysis of the epithelial-fiber interface (EFI) in primate lenses.. Exp Eye Res.

[5] Bassnett S, Winzenburger PA (2003). Morphometric analysis of fibre cell growth in the developing chicken lens.. Exp Eye Res.

[6] Goodenough DA (1992). The crystalline lens. A system networked by gap junctional intercellular communication.. Semin Cell Biol.

[7] Mathias RT, Rae JL, Baldo GJ (1997). Physiological properties of the normal lens.. Physiol Rev.

[8] Davy A, Gale NW, Murray EW, Klinghoffer RA, Soriano P (1999). Compartmentalized signaling by GPI-anchored ephrin-A5 requires the Fyn tyrosine kinase to regulate cellular adhesion.. Genes Dev.

[9] Holland SJ, Gale NW, Mbamalu G, Yancopoulos GD, Henkemeyer M (1996). Bidirectional signalling through the EPH-family receptor Nuk and its transmembrane ligands.. Nature.

[10] Takemoto M, Fukuda T, Sonoda R, Murakami F, Tanaka H (2002). Ephrin-B3-EphA4 interactions regulate the growth of specific thalamocortical axon populations in vitro.. Eur J Neurosci.

[11] Himanen JP, Chumley MJ, Lackmann M, Li C, Barton WA (2004). Repelling class discrimination: ephrin-A5 binds to and activates EphB2 receptor signaling.. Nat Neurosci.

[12] Poliakov A, Cotrina M, Wilkinson DG (2004). Diverse roles of eph receptors and ephrins in the regulation of cell migration and tissue assembly.. Dev Cell.

[13] Pasquale EB (2008). Eph-ephrin bidirectional signaling in physiology and disease.. Cell.

[14] Cooper MA, Son AI, Komlos D, Sun Y, Kleiman NJ (2008). Loss of ephrin-A5 function disrupts lens fiber cell packing and leads to cataract.. Proc Natl Acad Sci U S A.

[15] Shiels A, Bennett TM, Knopf HL, Maraini G, Li A (2008). The EPHA2 gene is associated with cataracts linked to chromosome 1p.. Mol Vis.

[16] Jun G, Guo H, Klein BE, Klein R, Wang JJ (2009). EPHA2 is associated with age-related cortical cataract in mice and humans.. PLoS Genet.

[17] Zhang T, Hua R, Xiao W, Burdon KP, Bhattacharya SS (2009). Mutations of the EPHA2 receptor tyrosine kinase gene cause autosomal dominant congenital cataract.. Human mutation.

[18] Kaul H, Riazuddin SA, Shahid M, Kousar S, Butt NH (2010). Autosomal recessive congenital cataract linked to EPHA2 in a consanguineous Pakistani family.. Molecular vision.

[19] Tan W, Hou S, Jiang Z, Hu Z, Yang P (2011). Association of EPHA2 polymorphisms and age-related cortical cataract in a Han Chinese population.. Molecular vision.

[20] Runge PE, Hawes NL, Heckenlively JR, Langley SH, Roderick TH (1992). Autosomal dominant mouse cataract (Lop-10). Consistent differences of expression in heterozygotes.. Investigative ophthalmology & visual science.

[21] Gong X, Agopian K, Kumar NM, Gilula NB (1999). Genetic factors influence cataract formation in alpha 3 connexin knockout mice.. Developmental genetics.

[22] Sandilands A, Wang X, Hutcheson AM, James J, Prescott AR (2004). Bfsp2 mutation found in mouse 129 strains causes the loss of CP49' and induces vimentin-dependent changes in the lens fibre cell cytoskeleton.. Experimental eye research.

[23] Alizadeh A, Clark J, Seeberger T, Hess J, Blankenship T (2004). Characterization of a mutation in the lens-specific CP49 in the 129 strain of mouse.. Investigative ophthalmology & visual science.

[24] Simirskii VN, Lee RS, Wawrousek EF, Duncan MK (2006). Inbred FVB/N mice are mutant at the cp49/Bfsp2 locus and lack beaded filament proteins in the lens.. Investigative ophthalmology & visual science.

[25] Cheng C, Xia CH, Li L, White TW, Niimi J (2008). Gap junction communication influences intercellular protein distribution in the lens.. Experimental eye research.

[26] Shestopalov VI, Bassnett S (2003). Development of a macromolecular diffusion pathway in the lens.. Journal of cell science.

[27] Pontoriero GF, Smith AN, Miller LA, Radice GL, West-Mays JA (2009). Co-operative roles for E-cadherin and N-cadherin during lens vesicle separation and lens epithelial cell survival.. Developmental biology.

[28] Leonard M, Zhang L, Zhai N, Cader A, Chan Y (2011). Modulation of N-cadherin junctions and their role as epicenters of differentiation-specific actin regulation in the developing lens.. Developmental biology.

[29] Xu L, Overbeek PA, Reneker LW (2002). Systematic analysis of E-, N- and P-cadherin expression in mouse eye development.. Experimental eye research.

[30] Kreslova J, Machon O, Ruzickova J, Lachova J, Wawrousek EF (2007). Abnormal lens morphogenesis and ectopic lens formation in the absence of beta-catenin function.. Genesis.

[31] Cain S, Martinez G, Kokkinos MI, Turner K, Richardson RJ (2008). Differential requirement for beta-catenin in epithelial and fiber cells during lens development.. Developmental biology.

[32] Straub BK, Boda J, Kuhn C, Schnoelzer M, Korf U (2003). A novel cell-cell junction system: the cortex adhaerens mosaic of lens fiber cells.. Journal of cell science.

[33] Martinez G, Wijesinghe M, Turner K, Abud HE, Taketo MM (2009). Conditional mutations of beta-catenin and APC reveal roles for canonical Wnt signaling in lens differentiation.. Investigative ophthalmology & visual science.

[34] Bassnett S, Wilmarth PA, David LL (2009). The membrane proteome of the mouse lens fiber cell.. Mol Vis.

[35] Frieden LA, Townsend TA, Vaught DB, Delaughter DM, Hwang Y (2010). Regulation of heart valve morphogenesis by Eph receptor ligand, ephrin-A1.. Developmental dynamics: an official publication of the American Association of Anatomists.

[36] Stephen LJ, Fawkes AL, Verhoeve A, Lemke G, Brown A (2007). A critical role for the EphA3 receptor tyrosine kinase in heart development.. Developmental biology.

[37] Bassnett S, Missey H, Vucemilo I (1999). Molecular architecture of the lens fiber cell basal membrane complex.. Journal of cell science.

[38] Frisen J, Yates PA, McLaughlin T, Friedman GC, O'Leary DD (1998). Ephrin-A5 (AL-1/RAGS) is essential for proper retinal axon guidance and topographic mapping in the mammalian visual system.. Neuron.

[39] Gong X, Li E, Klier G, Huang Q, Wu Y (1997). Disruption of alpha3 connexin gene leads to proteolysis and cataractogenesis in mice.. Cell.

[40] Okabe M, Ikawa M, Kominami K, Nakanishi T, Nishimune Y (1997). ‘Green mice’ as a source of ubiquitous green cells.. FEBS Lett.

[41] Sugiyama Y, Stump RJ, Nguyen A, Wen L, Chen Y (2010). Secreted frizzled-related protein disrupts PCP in eye lens fiber cells that have polarised primary cilia.. Developmental biology.

